# Invasive extramammary Paget's disease with lymph node metastases and high-grade B-cell lymphoma^⋆^

**DOI:** 10.1016/j.abd.2022.04.012

**Published:** 2023-02-17

**Authors:** Misato Ueda, Makoto Omori, Ayumi Sakai

**Affiliations:** Department of Plastic Surgery, Yodogawa Christian Hospital, Kunijima, Osaka, Japan

Dear Editor,

Extramammary paget’s disease (EMPD) has been associated with other several malignancies.^1^ The relationship between EMPD and secondary malignancies has been frequently reported. Due to increased risks of other malignancies, prolonged follow-up for EMPD and screening for malignancies are recommended. Patients with EMPD of the vulva and perianal regions have an increased risk of malignancies derived from the genitourinary and colorectal systems.^2^ However, other malignancies may also develop. Only a few cases of EMPD with lymphomas have been previously reported.^1^, ^3^ The authors present a case involving a patient who developed high-grade B-cell malignant lymphoma during a follow-up for invasive EMPD with lymph node metastases.

## Case report

**Figure 1:**
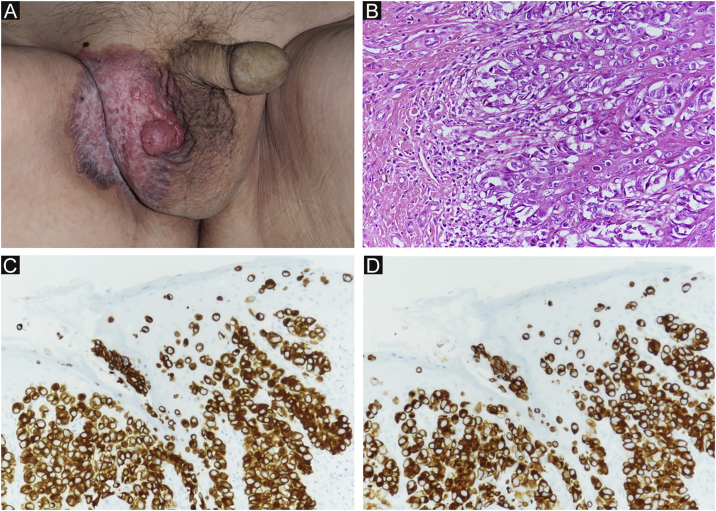
(A) Clinical findings of primary EMPD. A rash and elevated lesions are found in the right scrotum. (B) Primary EMPD: Tumors that grow individually or alveolarly are found in the basal layer of the epidermis. There is a part where infiltration into the dermis layer is observed. Tumor cells have pale-colored vesicles, and the nuclei are irregularly shaped and unevenly distributed. (Hematoxylin & eosin, ×200). (C) Positive CK7 immunostaining (immunohistochemical staining for CK7 × 200). (D) Positive CAM2.5 immunostaining (Immunohistochemical staining for CAM2.5, ×200).

**Figure 2:**
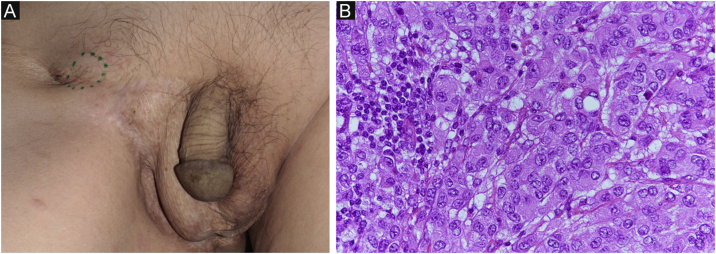
Clinical findings. A subcutaneous mass in the right inguinal area is observed. (A) Atypical cells with a pale cytoplasm proliferated rapidly, and the findings are similar to those of previous invasive Paget’s disease (Hematoxylin & eosin, ×400).

An 80-year-old man was referred to our department for *in situ* EMPD of the scrotal region (Fig. 1A). Preoperative Computed Tomography (CT) did not reveal metastases. We performed tumor resection with a margin of 10 mm and skin grafting. Pathological examination of the excised specimen revealed invasive EMPD (Fig. 1B‒D). However, both horizontal and vertical margins were negative. He underwent follow-up in our outpatient department. Approximately two years after surgery, right inguinal lymphadenopathy was observed (Fig. 2A), with no obvious cutaneous lesions. A lymph node biopsy revealed metastases of EMPD. Lymph node dissection was performed in the inguinal and external iliac artery areas. Histopathologically, 7/12 lymph nodes had evidence of invasive EMPD metastases (Fig. 2B). Postoperative Radiotherapy (RT) was administered; the patient received a total dose of 46 Gy.

One month after RT, CT revealed decreased intraperitoneal lymph nodes. However, lymphadenopathies to the para-aortic and left supraclavicular nodes were observed. He did not desire additional RT or systemic chemotherapy and opted for receiving supportive palliative treatment. Subsequently, peritoneal dissemination and multiple intra-abdominal lymph node metastases began to be observed.

**Figure 3:**
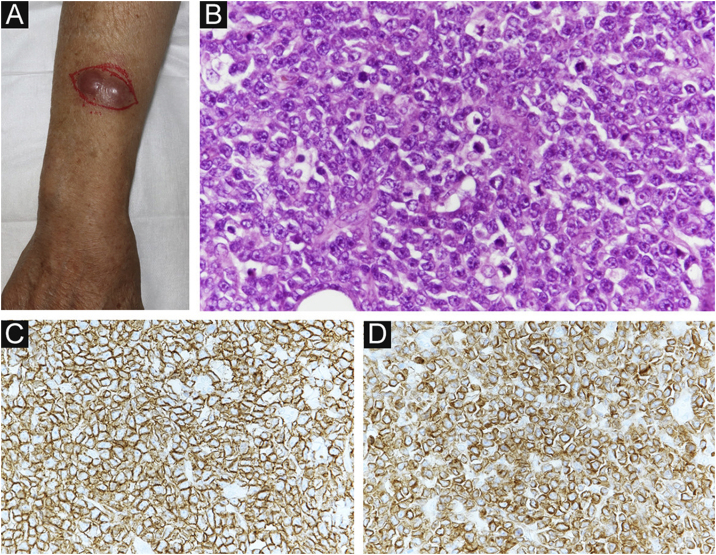
(A) Clinical findings: A painful subcutaneous tumor is observed in the right forearm. (B) Small round atypical cells are diffusely proliferating, showing a bottom-heavy appearance (Hematoxylin & eosin, ×200). (C) Positive CD20 immunostaining (Immunohistochemical staining for MYC × 400). (D) Positive CD79a immunostaining (immunohistochemical staining for BCL-2, ×400).

**Figure 4:**
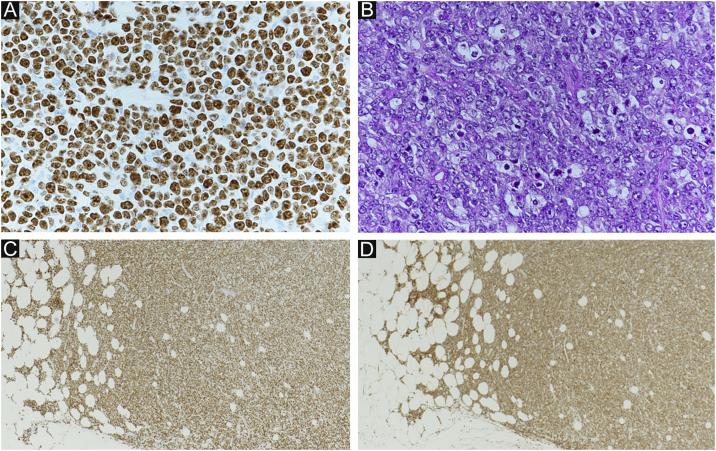
(A) Ki-67 index: 99%. (B) Starry sky (macrophages phagocytosed spallation is found in the proliferation of atypical lymphocytes). (A and B) showed high grade lymphoma. (C) Positive MYC immunostaining (immunohistochemical staining for MYC × 40). (D) Positive BCL-2 immunostaining (immunohistochemical staining for BCL-2, ×40). (C and D) showed findings of double expressor lymphoma.

**Figure 5:**
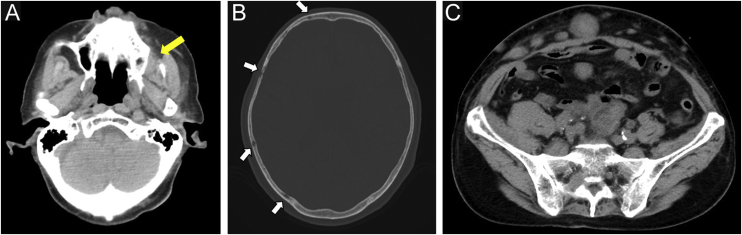
Computed tomography findings. (A) Soft tissue tumor in the left maxillary sinus (yellow arrow). (B) Osteolytic changes in the skull (white arrows). (C) Multiple abdominal subcutaneous and intraperitoneal metastases.

Four months after RT, a painful subcutaneous tumor was observed on his right forearm (Fig. 3A). The tumor was resected for pain relief. Histopathologically, the diffuse proliferation of small round atypical cells was noted (Fig. 3B). Both CD20 and CD79a, which are specific markers for B-lineage derivation, were positive by immunohistochemistry (Fig. 3C‒D). Ki-67 was significantly elevated at 99% (Fig. 4A). Moreover, findings of starry-sky appearance (macrophages phagocytosed spallation was found in the proliferation of atypical lymphocytes) (Fig. 4B) were found. These findings showed high-grade malignancy. Pathological examination confirmed a high-grade B-cell lymphoma. Immunohistochemistry showed positivity for both MYC and BCL-2, consistent with double-expressor lymphoma (Fig. 4C‒D). One week later, he was admitted to our department due to deteriorations in his general condition. CT revealed findings suggestive of maxillary sinus, skull, and multiple systemic lesions (Fig. 5). He received palliative treatment and died 12 days after admission.

## Discussion

The prognosis of EMPD, intraepidermal cancer, is favorable in most cases. However, the prognosis of invasive EMPD is poor, especially in cases of lymphovascular invasion.^4^ Lymph node metastases are an important prognostic factor.^5^

Other primary tumors may develop secondary to EMPD. The incidence of secondary malignancies is reportedly high (8.6%–42%).^2^, ^6^, ^7^, ^8^ Invasive EMPD is associated with a higher risk of secondary malignancies than EMPD *in situ*. Patients with invasive EMPD have a >50% higher risk of developing secondary malignancies following their original diagnosis.^1^ This risk remains significantly elevated over time. Therefore, thorough examination of other tumors during follow-up is recommended in patients with EMPD.^8^

It has been frequently reported that EMPD is associated with various malignant tumors.^1^, ^2^, ^8^, ^9^ In particular, it has been reported that there is an increased risk of secondary genitourinary and colorectal tumors in patients with EMPD of the vulvar and perianal regions.^2^

Kiltos et al.^9^ conducted a database analysis study in the United States and reported that gastrointestinal, breast and urogenital cancers are the most common secondary malignancies in patients with invasive vulvar Paget’s disease and that the risk of developing secondary malignancies is higher in these patients than in the standard population (controls). The authors also reported that the frequencies of secondary malignancies of the gastrointestinal tract, urinary tract, genital tract, blood, and skin are higher in patients with invasive vulvar EMPD than in the general population. Karam et al.^1^ analyzed the standardized prevalence ratio for the development of secondary malignancies after the initial diagnosis of invasive EMPD and reported that the observed to an expected ratio (observed rate/expected rate) for lymphoma was ≥1, although no significant difference was apparent. However, there are only a few reports of EMPD with lymphoma.^1^, ^3^ Moreover, no study has provided a detailed description.

In our case, lymphadenopathies extending from the right external iliac artery were considered as manifestations of metastases of invasive EMPD. However, the subsequent distant metastases were likely malignant lymphoma. The rapidly enlarging abdominal subcutaneous tissue, maxillary sinus, and skull bone were suggestive of malignant lymphomas.

In our case, immunostaining for MYC and BCL-2 showed positive findings. MYC/BCL-2 co-expression without underlying rearrangements is a new adverse prognostic indicator, termed double‐expressor lymphoma.^10^ This patient had an advanced malignancy, and Ki-67 was significantly elevated at 99%. The disease was highly malignant and progressed rapidly.

Invasive EPMD also metastasizes to the lymphatics and may be difficult to be distinguished from malignant lymphoma, as observed in our case. The diagnosis of high-grade B-cell malignant lymphoma was established for the first time based on a resected metastatic skin lesion specimen. This pathological condition significantly affects the treatment, prognosis, and systemic condition of patients.

We believe that patients with invasive EMPD require follow-up, considering the possibility of the development of malignant tumors that can influence the prognosis, in addition to genitourinary and colorectal cancers.

## Financial support

None declared.

## Authors’ contributions

Misato Ueda: Approval of the final version of the manuscript; design and planning of the study; drafting and editing of the manuscript; collection, analysis, and interpretation of data; effective participation in research orientation; intellectual participation in therapeutic conduct of studied cases; critical review of the literature; critical review of the manuscript.

Makoto Omori: Approval of the final version of the manuscript; design and planning of the study; drafting and editing of the manuscript; effective participation in research orientation; intellectual participation in therapeutic conduct of studied cases; critical review of the manuscript.

Ayumi Sakai: Approval of the final version of the manuscript; design and planning of the study; intellectual participation in therapeutic conduct of studied cases; critical review of the manuscript.

## Conflicts of interest

None declared.
